# Indirect calorimetry compared with predictive equations for resting energy expenditure in patients with intestinal failure

**DOI:** 10.1016/j.intf.2026.100374

**Published:** 2026-04-28

**Authors:** Nicola Wyer, Ellen Reynolds, Laura Norrie, Laura Woodend, Holly Richardson, Eleanor Ferguson

**Affiliations:** University Hospitals Coventry and Warwickshire NHS Trust, Clifford Bridge Road, Coventry CV2 2DX, United Kingdom

**Keywords:** Intestinal failure, Indirect calorimetry, Parenteral nutrition

## Abstract

**Background:**

Accurate assessment of energy requirements is essential for patients with intestinal failure (IF) to avoid complications associated with parenteral nutrition. Predictive equations are commonly used in clinical practice to estimate resting energy expenditure (REE), however, evidence supporting their accuracy in IF patients is limited. Indirect calorimetry (IC) is the gold standard for measuring REE but is not routinely available. This study evaluated the agreement between routinely used predictive methods in the UK and IC-measured REE in patients with IF.

**Material and methods:**

A service evaluation was conducted at a tertiary IF centre. Adult inpatients with IF had REE estimated using predictive equations based on PENG and ESPEN guidance, followed by IC measurement. Agreement between predicted and measured REE was assessed using mean bias, Bland-Altman analysis, and proportion of estimates within ±10% of IC values. Associations between prediction accuracy and IF aetiology, age, sex, and body mass index (BMI) were explored using ANOVA/linear regression.

**Results and conclusion:**

Indirect calorimetry was performed in 91 patients (mean age 55 ± 17.4 years, mean BMI 21.6 ± 5.8 kg/m^2^). Mean measured REE was 1449 ± 297 kcal/day. Predictive equations showed poor agreement with IC at an individual level, with wide limits of agreement. Mean bias was + 18.6 kcal/day (-585 to +622 kcal/day) for PENG and + 94.9 kcal/day (-620 to +809 kcal/day) for ESPEN. Only 33.7 (PENG) and 30.4% (ESPEN) of estimates were within ±10% of measured REE. Predictive equations are inaccurate in IF patients, supporting routine use of IC to enable precise, individualised energy prescription.

## Introduction

Intestinal failure (IF) occurs when the gastrointestinal tract is unable to absorb adequate nutrition and fluids to sustain health either through oral dietary intake or enteral feeding. When intestinal failure occurs, parenteral nutrition (PN) is required to meet patients’ nutritional needs. Accurate assessment of energy requirements is essential in this population as PN is associated with higher complication rates compared to other routes of nutrition support, making accurate nutrient provision essential to prevent over or under feeding [1], [2].

In clinical practice, predictive equations are commonly used to estimate energy requirements for PN. However, these equations are often inaccurate, and there is limited evidence supporting their use in patients with intestinal failure. International guidelines for energy provision vary considerably [3]. Energy requirements can be influenced by multiple factors including clinical condition, nutritional status, fat free mass, and physical activity level. In patients with IF, predictive equations are particularly challenging to use as they are predominantly weight-based, and IF patients frequently experience fluid balance disturbances affecting weight accuracy.

Indirect calorimetry (IC) is considered the gold standard for measuring resting energy expenditure (REE), enabling individualised and precise energy prescription [4]. Despite its accuracy and recommendation for use in patients with IF [5] it is not routinely used in clinical practice due to cost and resource requirement. As a result, predictive equations remain the most used method, due to their accessibility and zero cost, despite their known poor accuracy [4], [6], [7].

Research directly comparing predictive equations with IC-measured REE in patients with intestinal failure is limited, although a larger evidence base exists within critical care populations [3], [8], [9], [10]. Greater understanding of the agreement between estimated and measured energy requirements in IF is essential to ensure nutritional care is optimised and PN-related complications are minimised.

The aim of this cohort study was to evaluate the difference between REE targets derived from predictive equations and measured REE obtained by indirect calorimetry in patients managed at a tertiary intestinal failure centre. Specific objectives were to:

• Quantify the difference between measured REE and REE predicted using standard equations.
• Identify clinical characteristic associated with larger discrepancies between measured and predicted REE.
• Assess the proportion of patients whose energy requirements are under or overestimated when predictive equations are used.

## Material and methods

Dietitian-led indirect calorimetry was introduced to an intestinal failure service in July 2023. A service evaluation was conducted using data collected between July 2023 and October 2025 from the single site. Patients had assessment of energy requirements using predictive equations during routine Dietitian review, [5], [11], [12], [13]. Two predictive equation methods were used. The first method (PENG [11]) is widely used in UK dietetic practice and is a stepped approach to determining nutritional status, body mass index (BMI) and uses a collection of evidence based resources to guide choice of REE calculation (kcal/kg), depending on the predominant clinical condition e.g. gastrointestinal surgery, Crohn’s disease. Specific guidance is also provided for when BMI is < 18.5 kg/m^2^ or > 30 kg/m^2^. The second method (ESPEN [5], [12], [13]) is a direct kcal/kg method, with kcal/kg value determined by how acutely unwell the patient was. During assessment, the Dietitian determined if the patient was clinically suitable for indirect calorimetry (Table 1), and following patient consent, indirect calorimetry was performed at an agreed time within 24 h of assessment using a validated portable device (Q-NRG+ COSMED), by a trained Dietitian.

**Table 1:** Inclusion and exclusion criteria.

**Inclusion**	**Exclusion**
Diagnosis of type 2/3 intestinal failure	Unstable medical condition
Inpatients	End of life care where nutrition support no longer appropriate
Adults ≥ 18 years of age	Oxygen dependant
Clinically stable	Inability to tolerate IC (e.g. agitation, confusion, claustrophobia)

Patients were given instruction to rest for at least 30 min prior to measurement and activity such as physiotherapy was avoided. Measurements were taken at least 2 h after mealtimes or PN, where applicable, as most patients were unable to consume oral intake. Measurements were taken with patients in a supine position, using canopy hood mode as patients were spontaneously breathing, and canopy mode has been demonstrated to be superior at achieving steady state compared to using a face mask [14]. Resting energy expenditure (REE) was recorded for a 5-minute period once steady state was achieved, defined as variation of VO_2_ and VCO_2_ ≤ 10%.

Data on age, sex, weight, body mass index (BMI) and IF aetiology was collected during dietetic assessment. Energy requirements calculated using predictive equations [5], [11], [12], [13] and IC measurement were recorded.

Differences between methods were analysed using mean absolute difference and mean percentage difference between measured REE and predictive values. Agreement was classified as accurate where predicted values were within ±10% of measured values. Overestimation was defined as predictive values > 10% above measured REE, and underestimation as predictive values < 10% below measured REE.

Bland-Altman plots [15] were used to assess agreement between IC-measured REE and predicted values. Bias was calculated as the mean difference (kcal/day) between methods, and limits of agreement were defined as the mean difference ±1.96 SD. One-way ANOVA / linear regression was used to assess whether differences varied according to IF aetiology, BMI, age, and sex.

This study was classified as a service evaluation of routine clinical practice. Under the UK Health Departments’ Governance Arrangements for Research Ethics Committees (GAfREC), formal NHS Research Ethics Committee review was not required because the project did not constitute research [16]. The project received local service evaluation approval (SE0428).

## Results

### Patient characteristics

Indirect calorimetry measurement was performed on 91 inpatients at a single point of time between July 2023 and October 2025, see Table 2 for patient characteristics. Patients had type 2 or type 3 intestinal failure and had required parenteral nutrition for > 28 days. 37 patients were excluded due to unstable medical condition (n = 14), oxygen dependence (n = 15) or assessed at being unsuitable for measurement due to tolerance concerns (n = 8).

**Table 2:** Patient characteristics.

**Characteristic**	**Patient group**
**Sex N = (%)**	
Male Female	37 (40.7%) 54 (59.3%)
**Age (year)**	
Mean (SD) Range	55 ± 17.4 18–82
**Weight (kg)**	
Mean (SD) Range	61.4 ± 17.0 30.6–114.3 kg
**BMI (kg/m)2**	
Mean (SD) Range	21.6 ± 5.8 11.8–43.5
**IF pathophysiological classification**[17]	
Short bowel Intestinal fistula Intestinal dysmotility Mechanical obstruction Extensive small bowel mucosal disease	28 (30.7%) 14 (15.4%) 25 (27.5%) 20 (22.0%) 4 (4.4%

### Predictive equations compared to indirect calorimetry

Measured REE ranged from 839 to 2346 kcal/day with a mean of 1449 ± 297 kcal/day. Mean measured REE was 1361 ± 272 kcal/day for females, and 1578 ± 289 kcal/day for males.

Estimated REE using PENG predictive methods ranged from 780 to 2345 kcal/day with a mean of 1467 ± 309 kcal/day. Mean estimated REE was 1368 ± 280 kcal/day for females, and 1612 ± 296 kcal/day for males.

Estimated REE using ESPEN predictive methods ranged from 780 to 2857 kcal/day with a mean of 1534 ± 415 kcal/day. Mean estimated REE was 1452 ± 398 kcal/day for females, and 1677 ± 409 kcal/day for males.

**Fig. 1:**
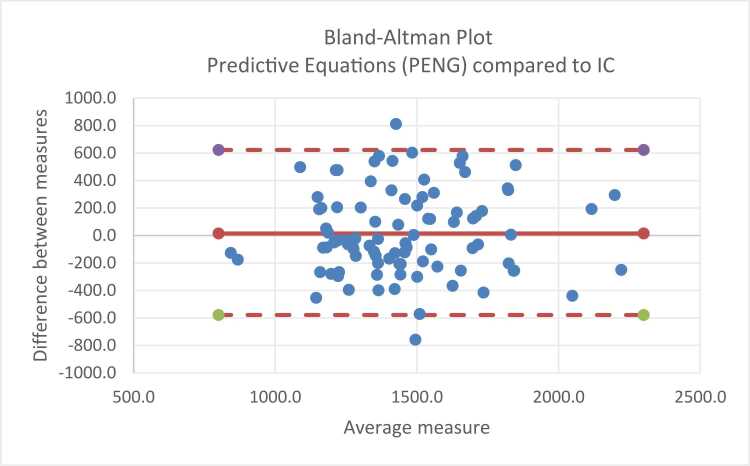
Bland-Altman Plot PENG methods of estimating REE compared to indirect calorimetry.

Bland-Altman analysis (Fig. 1) demonstrated a small mean bias of + 18.6 kcal/day for PENG predictive methods compared with indirect calorimetry. However, limits of agreement were wide (-585 to +622 kcal/day) indicating substantial variability and poor agreement of methods at an individual patient level.

**Fig. 2:**
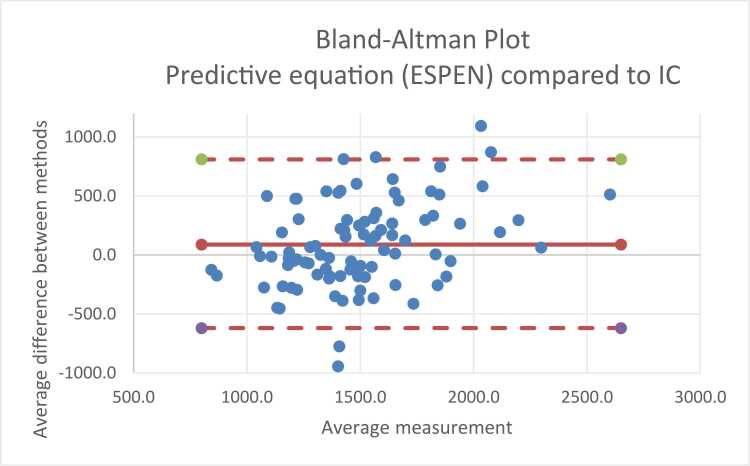
Bland-Altman Plot ESPEN predictive equation compared to indirect calorimetry.

Bland-Altman analysis (Fig. 2) for ESPEN predictive methods showed a larger mean bias of + 94.9 kcal/day with wide limits of agreement (-620 to +809 kcal/day), again demonstrating poor individual patient level agreement.

Only 33.7% of patients had estimated energy requirements within ±10% of measured REE when using PENG methods, compared with 30.4% when using ESPEN methods.

### IF aetiology, sex, and age

Differences between PENG predictive methods and measured REE (Table 3) were not associated with IF aetiology (F(4,86) = 0.47, p = 0.759), with aetiology explaining only 2.1% of variance (R^2^ = 0.021). In contrast, ESPEN predicted values differed between groups (F(4,86) = 4.42, p = 0.003) accounting for 17.1% of variance (R^2^ = 0.171). ESPEN estimates were significantly higher relative to indirect calorimetry in patients with intestinal fistula (mean difference +449 kcal/day, p = 0.022) and mechanical obstruction (+473 kcal/day, p = 0.013), with no significant differences observed for intestinal dysmotility or short bowel syndrome. No significant differences in accuracy of predictive equations method were observed across age groups or sex.

**Table 3:** Factors associated with differences between predictive equations and measured REE in patients with intestinal failure.

**Factor**	**PENG**		**ESPEN**	**Key interpretation**
Age		No association p > 0.05	No association p > 0.05	Age does not influence prediction error
Sex		No association p > 0.05	No association p > 0.05	Sex does not influence prediction error
IF aetiology		No association F(4,86) = 0.47, p = 0.759 R^2^ = 0.021	Significant association F(4,86) = 4.42, p = 0.003 R^2^ = 0.171	ESPEN significantly higher in intestinal fistula (p = 0.022) and mechanical obstruction (p = 0.013)
			

### Body mass index (BMI)

When stratified by BMI category, PENG derived estimates showed no significant bias across groups. In contrast, ESPEN derived estimates demonstrated underestimation in those with BMI < 18.5 kg/m^2^, with progressively increasing overestimation as BMI increased (Table 4). This was despite standard methods for weight adjustment for BMI > 30 kg/m^2^ being followed during calculations.

**Table 4:** ESPEN and PENG compared to IC across BMI categories.

**BMI**	**Number of patients (n)**		**PENG – IC mean difference** **(95% CI), kcal/day**	**ESPEN – IC mean difference** **(95% CI), kcal/day**
< 18.5 kg/m^2^		26	-63 (-140 to +15)	-162 (-292 to −32)
18.5–24.9 kg/m^2^		46	+ 63 (-3 to +130)	+ 108 (26–190)
25–29.9 kg/m^2^		10	+ 138 (-38 to +313)	+ 237 (27–447)
≥ 30 kg/m^2^		9	-107 (-298 to +84)	+ 612 (368–856)

## Discussion

This service evaluation of patients (n = 91) with intestinal failure demonstrates poor agreement between predictive equations and measured resting energy expenditure using indirect calorimetry at an individual patient level. Although mean bias was relatively small (+18.6 kcal/day for PENG and +94.9 kcal/day for ESPEN), wide limits of agreement indicated substantial individual patient variability. Overall, < 35% of predictive estimates were within ±10% of measured REE. Although this study showed a higher level of inaccuracy with ESPEN methods in intestinal fistula, mechanical obstruction and those with increasing BMI, the overall patient numbers are low, so should be viewed with caution.

Variation between measured and predicted REE may be attributable to various factors including co-morbidities, altered body composition, sarcopenia, and fluid balance disturbances. The decision-making by clinicians using predictive equations will also influence accuracy. Determining which equation to use from those available can be challenging, especially when evidence may relate to a single clinical condition, whereas in practice there is increasing prevalence of poly-morbidity. Where multiple conditions are present and interacting, it may be difficult to determine which factor to prioritise, as evidence is lacking on how combined conditions may influence energy expenditure. As a result, predictive equations risk being overly simplistic estimates in the presence of highly heterogeneous metabolic states.

The contribution of fat mass to resting energy expenditure is substantially lower than that of fat-free mass, reflecting the lower metabolic activity of adipose tissue. Therefore, predictive equations, derived from total body weight may inadequately estimate energy requirements, as they do not account for variation in body composition and the different metabolic contribution of lean and adipose tissue. Standard methods used in clinical practice to adjust measured total body weight for high BMI, or fluid disturbance e.g. adjusted body weight, did not correct sufficiently for overestimation in patients with higher BMI using ESPEN methods, confirming the limitations of weight-based predictive equations in this sample [18], [19].

It is important to recognise the inconsistency in terminology across the literature when describing the estimation of energy requirements. Some sources define energy targets in relation to resting energy expenditure (REE), which does not account for physical activity, whereas others recommend approaches intended to estimate total energy expenditure (TEE). The distinction is often not explicitly stated. This lack of clarity has the potential to result in misapplication of equations in clinical practice, further complicating the use and interpretation of predictive equations creating variability in practice. Both PENG [11] and ESPEN [5], [12], [13] recognise that methods used to estimate energy requirements can be inaccurate and should be used only where indirect calorimetry is not available. They also recommend that patient response to treatment should be monitored carefully.

In this complex patient population, inaccurate energy provision may contribute to complications [1], [20], [21], [22]. Overfeeding, particularly in patients with obesity, may increase the risk of intestinal failure associated liver disease (IFALD), and metabolic complications such as hyperglycaemia [20], [21], [22]. Underfeeding malnourished patients may delay nutritional rehabilitation and prolong hospital length of stay. This emphasises the importance of precision nutrition and individualised assessment of energy requirements in intestinal failure.

Strengths of this study include the sample having a range of patient characteristics and use of UK standard predictive methods. Limitations include the single-centre, cross-sectional design, with single time point measurement. Patients unable to tolerate IC were excluded, potentially limited generalisability.

These results support the routine use of indirect calorimetry in clinical practice where resources are available. Where IC is not available, and predictive equations relied upon, clinicians must be aware of the potential wide individual patient variation and monitor progress closely.

Future research should focus on evaluating the impact of IC guided nutrition treatment plans on clinical outcomes, including nutritional status, metabolic complications, and hospital length of stay and whether it is possible to develop IF specific predictive equations for this heterogeneous patient group.

## Conclusion

Predictive equations demonstrate significant inaccuracy at the individual patient level when compared with indirect calorimetry-measured resting energy expenditure in patients with intestinal failure. Given the increased risk of complications associated with iatrogenic under or overfeeding in this patient group, reliance on predictive equations is insufficient.

Where available, indirect calorimetry should be routinely used to guide energy prescription in intestinal failure. In settings where IC is unavailable, predictive equations should be used with caution, particularly in patients with obesity, there should be awareness of the wide variability in predictive accuracy at individual patient level, and clinical parameters should be monitored closely.

## CRediT authorship contribution statement

**Laura Norrie:** Writing – review & editing, Investigation, Data curation. **Ellen Reynolds:** Writing – review & editing, Project administration, Formal analysis, Data curation. **Nicola Wyer:** Writing – review & editing, Writing – original draft, Validation, Supervision, Software, Resources, Project administration, Methodology, Investigation, Formal analysis, Data curation, Conceptualization. **Eleanor Ferguson:** Writing – review & editing, Project administration, Investigation, Data curation. **Holly Richardson:** Writing – review & editing, Project administration, Investigation, Data curation. **Laura Woodend:** Writing – review & editing, Project administration, Investigation, Data curation.

## Patient's/Guardian's consent

Not applicable.

## Ethical clearance

As this was a service evaluation GafREC approval was obtained (SE0428 24/10/25). Research ethics committee approval was not required.

## Funding

This research did not receive any specific grant from funding agencies in the public, commercial or not-for-profit sectors.

## Declaration of Competing Interest

The authors declare that they have no known competing financial interests or personal relationships that could have appeared to influence the work reported in this paper.

## References

[1] Ukleja A., Romano M.M. (2007). Complications of parenteral nutrition. Gastroenterol Clin N Am.

[2] Lappas B.M., Patel D., Kumpf V., Adams D.W., Seidner D.L. (2018). Parenteral nutrition: indications, access, and complications. Gastroenterol Clin N Am.

[3] Ławiński M., Singer P., Gradowski Ł, Gradowska A., Bzikowska A., Majewska K. (2015). Predicted versus measured resting energy expenditure in patients requiring home parenteral nutrition. Nutrition.

[4] Delsoglio M., Achamrah N., Berger M.M., Pichard C. (2019). Indirect calorimetry in clinical practice. J Clin Med.

[5] Klek S., Forbes A., Gabe S., Holst M., Wanten G., Irtun Ø. (2016). Management of acute intestinal failure: a position paper from the European Society for Clinical Nutrition and Metabolism (ESPEN) Special Interest Group. Clin Nutr.

[6] Jésus P., Achamrah N., Grigioni S., Charles J., Rimbert A., Folope V. (2015). Validity of predictive equations for resting energy expenditure according to the body mass index in a population of 1726 patients followed in a nutrition unit. Clin Nutr.

[7] Boullata J., Williams J., Cottrell F., Hudson L., Compher C. (2007). Accurate determination of energy needs in hospitalized patients. J Am Diet Assoc.

[8] Oshima T., Berger M.M., De Waele E., Guttormsen A.B., Heidegger C.P., Hiesmayr M. (2017). Indirect calorimetry in nutritional therapy. A position paper by the ICALIC study group. Clin Nutr.

[9] Moonen H.P.F.X., Beckers K.J.H., van Zanten A.R.H. (2021). Energy expenditure and indirect calorimetry in critical illness and convalescence: current evidence and practical considerations. J Intensive Care.

[10] Duan J.Y., Zheng W.H., Zhou H., Xu Y., Huang H. Bin (2021). Energy delivery guided by indirect calorimetry in critically ill patients: a systematic review and meta-analysis. Crit Care.

[11] PENG (2018). A pocket guide to clinical nutrition.

[12] Pironi L., Boeykens K., Bozzetti F., Joly F., Klek S., Lal S. (2020). ESPEN guideline on home parenteral nutrition. Clin Nutr.

[13] Pironi L., Arends J., Bozzetti F., Cuerda C., Gillanders L., Jeppesen P.B. (2016). ESPEN guidelines on chronic intestinal failure in adults. Clin Nutr.

[14] Dupertuis Y.M., Delsoglio M., Hamilton-James K., Berger M.M., Pichard C., Collet T.H. (2022). Clinical evaluation of the new indirect calorimeter in canopy and face mask mode for energy expenditure measurement in spontaneously breathing patients. Clin Nutr.

[15] Martin Bland J., Altman D.G. (1986). Statistical methods for assessing agreement between two methods of clinical measurement. Lancet.

[16] Health Research Authority (2021). Governance arrangements for research ethics committees: 2020 edition.

[17] Pironi L., Arends J., Baxter J., Bozzetti F., Peláez R.B., Cuerda C. (2015). ESPEN endorsed recommendations: definition and classification of intestinal failure in adults. Clin Nutr.

[18] Madden A.M., Smith S. (2016). Body composition and morphological assessment of nutritional status in adults: a review of anthropometric variables. J Hum Nutr Diet.

[19] Thibault R., Abbasoglu O., Ioannou E., Meija L., Ottens-Oussoren K., Pichard C. (2021). ESPEN guideline on hospital nutrition. Clin Nutr.

[20] Sharkey L., Committee B.I.F.A. (2022).

[21] Sobotka L., Camilo M.E. (2009). Basics in clinical nutrition: metabolic complications of parenteral nutrition. E-SPEN.

[22] Davila J., Konrad D. (2017). Metabolic complications of home parenteral nutrition. Nutr Clin Pract.

